# COVID-19-Pandemie und die psychische Gesundheit in Deutschland: Verlauf, resiliente und vulnerable Gruppen

**DOI:** 10.1007/s00115-025-01824-8

**Published:** 2025-04-03

**Authors:** J. M. Stoffers-Winterling, H. F. Wiegand, J. Broll, S. K. Schäfer, K. Adorjan, O. Tüscher, K. Lieb

**Affiliations:** 1 Leibniz-Institut für Resilienzforschung (LIR) gGmbH, Wallstr. 7, 55122 Mainz, Deutschland; 2 Klinik für Psychiatrie und Psychotherapie, Universitätsmedizin Mainz, Mainz, Deutschland; 3 Universitätsklinik für Psychiatrie, Psychotherapie und Psychosomatik, Universitätsmedizin Halle, Halle, Deutschland; 4 Klinische Psychologie, Psychotherapie und Diagnostik, Technische Universität Braunschweig, Braunschweig, Deutschland; 5 Universitätsklinik für Psychiatrie und Psychotherapie, Universität Bern, Bern, Schweiz; 6 Klinik für Psychiatrie und Psychotherapie, Universitätsklinikum LMU München, München, Deutschland

**Keywords:** Risikogruppen, Resilienzfaktoren, Vulnerabilität, Kognitive Flexibilität, Soziale Unterstützung, Risk groups, Resilience factors, Vulnerability, Cognitive flexibility, Social support

## Abstract

**Hintergrund:**

Die COVID-19(„coronavirus disease 2019“)-Pandemie stellte die bisher wohl größte gesundheitliche Krise des 21. Jahrhunderts dar. Sie bedeutete für viele Menschen eine andauernde Exposition gegenüber psychisch relevanten Stressoren bei gleichzeitig eingeschränkten Bewältigungsmöglichkeiten.

**Ziel der Arbeit:**

Die vorliegende Übersicht hat zum Ziel, den aktuellen Kenntnisstand zum Verlauf der psychischen Gesundheit in Deutschland während der COVID-19-Pandemie zusammenfassend darzustellen.

**Material und Methoden:**

Anhand longitudinaler und wiederholt-querschnittlicher Erhebungen werden wesentliche Erkenntnisse zusammengefasst und Vulnerabilitäts- und Resilienzfaktoren herausgearbeitet.

**Ergebnisse:**

Berichtet werden für weite Teile der Bevölkerung zumindest vorübergehende Beeinträchtigungen des psychischen Wohlbefindens im Sinne einer erhöhten Angstsymptomatik und Depressivität sowie einer verringerten Lebenszufriedenheit, insbesondere bei Frauen und Kindern bzw. Jugendlichen. Gleichzeitig wurden in der Mehrzahl der Fälle resiliente Verläufe beobachtet, d. h. den meisten Personen gelang es während der Pandemie, ihre psychische Gesundheit aufrechtzuerhalten. Als Vulnerabilitätsfaktoren gelten neben einer weiblichen Geschlechtszugehörigkeit und einem jüngeren Alter auch finanzielle Schwierigkeiten. Als wichtige Resilienzfaktoren zeigen sich dagegen ein positiver Bewertungsstil, kognitive Flexibilität, soziale Unterstützung, Selbstwirksamkeitserleben und, auf gesellschaftlicher Ebene, sozialer Zusammenhang und Vertrauen in Institutionen.

**Diskussion:**

Die identifizierten Vulnerabilitäts- und Resilienzfaktoren bieten konkrete Ansatzpunkte zur Förderung der „pandemic preparedness“.

## Abstract (en)

**Background:**

The COVID-19 pandemic has probably been the biggest health crisis of the twenty-first century to date. For many people it meant prolonged exposure to psychologically relevant stressors and simultaneously limited coping possibilities.

**Aim of the study:**

The aim of this review is to present a summary of the current state of knowledge on the course of mental health in Germany during the COVID-19 pandemic.

**Material and methods:**

Using longitudinal and repeated cross-sectional surveys, key findings are summarized and vulnerability and resilience factors are identified.

**Results:**

Large proportions of the German population reported at least temporary effects on their mental well-being in the sense of increased anxiety and depression symptoms as well as reduced life satisfaction, particularly among women, children and adolescents. At the same time, resilient courses were observed in most cases, i.e., most people managed to maintain their mental health during the pandemic. Vulnerability factors included female gender, younger age and financial difficulties. In contrast, a positive appraisal style, cognitive flexibility, social support, self-efficacy beliefs and, at a societal level, social cohesion and institutional trust were reported as important resilience factors.

**Discussion:**

The vulnerability and resilience factors identified offer concrete starting points for promoting pandemic preparedness.

## Hintergrund

Während zu Beginn und den Hochprävalenzphasen der COVID-19(„coronavirus disease 2019“)-Pandemie in den Jahren 2020 und 2021 v. a. die somatische Perspektive im Fokus der öffentlichen Diskussion stand, weitete sich im mittelfristigen Verlauf der Fokus auch auf psychische Konsequenzen der Pandemie. Nachdem es anfänglich, insbesondere aufgrund der Verwendung hochsensitiver Screeninginstrumente für psychische Erkrankungen ohne weitere Diagnosesicherung, zu einer Überschätzung psychischer Erkrankungsraten kam, wird nun mit einigem zeitlichen Abstand sichtbar, welche Auswirkungen tatsächlich durch wissenschaftliche Evidenz nachweisbar sind, welche Gruppen besonders betroffen waren und welche Lehren daraus für künftige Krisenlagen gezogen werden können. Hier zeigen sich individuelle und gesellschaftliche Resilienzfaktoren, die schädliche Effekte abfedern können.

Die vorliegende Übersichtsarbeit bildet den Auftakt zum Minischwerpunkt „Psychische Gesundheit und psychiatrisch-psychotherapeutische Versorgung in der COVID-19-Pandemie“ und soll den aktuellen Kenntnisstand zum Verlauf der psychischen Gesundheit in Deutschland während der COVID-19-Pandemie, zu vulnerablen Gruppen und individuellen sowie gesellschaftlichen Resilienzfaktoren darstellen.

## Verlauf der psychischen Gesundheit während der Covid-19-Pandemie

*Weltweit* wurden in der frühen Pandemiephase in der Allgemeinbevölkerung Effekte auf die psychische Gesundheit beobachtet. Ein großes Umbrella-Review zeigt bei erheblicher Heterogenität der Einzelstudien in der Frühphase der Pandemie positive Screeningergebnisse für depressive Erkrankungen bei 16–48 % der Allgemeinbevölkerung, für Angststörungen bei 15–47 % und für posttraumatische Belastungsstörungen bei 9–33 % [29]. Das tatsächliche Vorhandensein einer klinisch relevanten Diagnose wurde dabei i. d. R. gesichert. Tatsächliche Funktionseinschränkungen, Differenzialdiagnostik oder Verlauf wurden nicht beurteilt, sodass hier von einem hohen Anteil falsch-positiver Ergebnisse auszugehen ist.

**Abb. 1:**
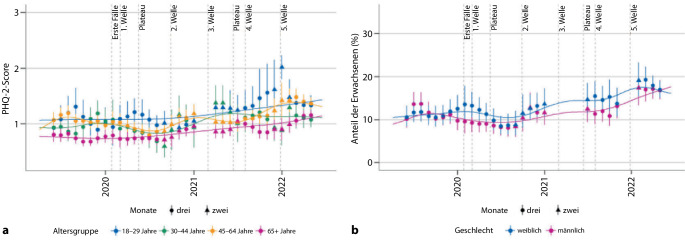
**a** Verlauf der Depressivität nach Altersgruppen (Patient Health Questionnaire‑2, PHQ-2). (Abb. aus [19], © 2023 Mauz, Walther, Junker, Kersjes, Damerow, Eicher, Hölling, Müters, Peitz, Schnitzer und Thom; CC BY, http://creativecommons.org/licenses/by/4.0/). **b** Anteil der Erwachsenen mit positivem Depressivitätsscreening (PHQ-2), nach Männern und Frauen getrennt. (Abb. aus [19], © 2023 Mauz, Walther, Junker, Kersjes, Damerow, Eicher, Hölling, Müters, Peitz, Schnitzer und Thom; CC BY, http://creativecommons.org/licenses/by/4.0/)

*Deutsche* Längsschnittdaten, wie diejenigen der bevölkerungsrepräsentativen Studie „Gesundheit in Deutschland aktuell“ (GEDA), zeigten dabei zu Beginn der COVID-19-Pandemie im April/Mai 2020 gegenüber dem entsprechenden Vorjahreszeitraum eine Abnahme der *Depressivität*, die sich nach Abklingen der ersten Pandemiewelle bis zum Sommer 2020 fortsetzte. Im Zuge der ersten Winterwelle ab Herbst 2020 gab es einen erneuten Anstieg, wobei das Depressivitätsniveau im Anschluss gegenüber dem vorpandemischen Vergleichszeitraum erhöht blieb und zwischen 2021 und 2022 weiterhin anstieg ([19], Abb. 1a). Auch der Anteil der Personen mit einem klinisch relevanten Depressivitätsscreening erhöhte sich entsprechend [19], analog zu Beobachtungen aus anderen westlichen Ländern [23, 29].

**Abb. 2:**
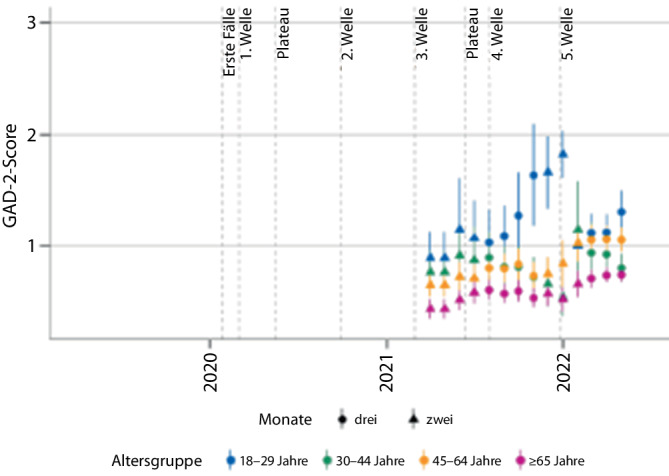
Belastung durch Angstsymptome in Deutschland – bevölkerungsrepräsentative Erhebungen des Robert-Koch-Instituts (GEDA[Gesundheit in Deutschland aktuell]- und COVIMO[COVID-19-Impfquoten-Monitoring in Deutschland]-Surveys), Mittelwerte der Generalized Anxiety Disorder Scale‑7 (GAD-7) nach Altersgruppen getrennt. (Abb. aus [19], © 2023 Mauz, Walther, Junker, Kersjes, Damerow, Eicher, Hölling, Müters, Peitz, Schnitzer und Thom; Creative Commons Attribution License; CC BY, http://creativecommons.org/licenses/by/4.0/)

Eine bevölkerungsrepräsentative Studie weist auf eine rasche Habituation der *Belastung durch Angstsymptome* in den ersten Monaten der Pandemie zwischen April bis Juni 2020 hin [17]. Seit Frühjahr/Sommer 2021 ist jedoch ein erneuter Anstieg zu beobachten, der besonders stark bei jungen Erwachsenen im Alter von 18 bis 29 Jahren sowie Gruppen mit mittlerem und hohem Bildungsgrad ausfällt ([19], Abb. 2).

Betrachtet man den Verlauf des allgemeinen *subjektiven Wohlbefindens* und der *Lebenszufriedenheit*, so zeigen repräsentative Studien aus Deutschland in den ersten Monaten der Pandemie zunächst einen gegenüber dem Vorjahr stabilen Verlauf sowie eine leichte Abnahme im späteren Verlauf bis Februar 2021, der bei differenzierter Betrachtung jedoch deutlich divergiert [18]: In der Frühphase der Pandemie zeigte sich eine Zunahme der Lebenszufriedenheit bei Personen mit niedriger Bildung und niedrigem Einkommen, jedoch eine gegensätzliche Entwicklung bei Menschen mit hohem Bildungsgrad und hohem Einkommen [7]. Im Laufe der Pandemie nahm sie insbesondere bei Selbständigen und Frauen weiter ab, während das allgemeine Wohlbefinden bei Menschen, die allein wohnten, zunahm und bei Paaren mit Kindern abnahm [7]. Im Mittel findet der Eurobarometer-Survey im Langzeitverlauf zwischen November 2019 und Juli 2022 jedoch keine substanzielle Änderung der Lebenszufriedenheit in Deutschland, wobei die jährlichen Erhebungen zwischen 3,25 und 3,20 Punkten auf der Eurobarometerskala (Range: 1–4) schwanken [6].

## Vulnerable Gruppen

**Abb. 3:**
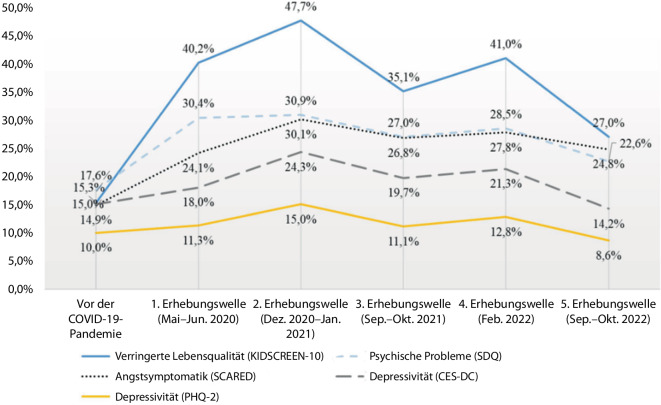
Verlauf der mentalen Gesundheit von Kindern und Jugendlichen im Alter von 7 bis 17 Jahren in Deutschland – Ergebnisse der repräsentativen COPSY-Studie (Covid-19 and Psychological Health). (Abb. aus [21], © 2023 Ravens-Sieberer, Devine, Napp, Kaman, Saftig, Gilbert, Reiß, Löffler, Simon, Hurrelmann, Walper, Schlack, Hölling, Wieler und Erhart; Creative Commons Attribution License; CC BY, http://creativecommons.org/licenses/by/4.0/)

Konsistent zeigt sich sowohl in Deutschland als auch international eine erhöhte Vulnerabilität bei Kindern und Jugendlichen. Die repräsentative deutsche COPSY(Covid-19 and Psychological Health)-Längsschnittstudie fand bei *Kindern und Jugendlichen* eine Verschlechterung der Lebensqualität, vermehrte emotionale und Verhaltensprobleme sowie eine erhöhte Belastung durch Angstsymptome, welche im Verlauf jeweils nach Höchststand zum Jahreswechsel 2020/21 und im Februar 2022 das präpandemische Ausgangsniveau nicht mehr erreichen, mit Ausnahme der Depressivität ([21]; Abb. 3). Als Prädiktoren für ungünstigere Verläufe wurden dabei weibliche Geschlechtszugehörigkeit, ein jüngeres Alter innerhalb der Gruppe der 7‑ bis 17-Jährigen sowie geringere familiäre Kohäsion identifiziert [31]. Eine internationale Übersichtsarbeit fand als weitere Einflussfaktoren den sozioökonomischen Status, den elterlichen Bildungsstatus, die präpandemische somatische und psychische Gesundheit, die Fähigkeit zur Selbstregulation, soziale Unterstützung, Einsamkeit, gesundheitsbezogene Sorgen sowie konsistente Routinen und Tagesstruktur, aber auch familiäre Faktoren wie die psychische Gesundheit der Eltern, das Erziehungsverhalten und das familiäre Funktionsniveau [30].

Bei *Menschen höheren Alters* findet sich im Mittel lediglich eine geringe Zunahme an psychischer Belastung allgemein, von Depressivität, Belastungen durch Angstsymptome, Einsamkeit (jeweils signifikante Effektstärken zwischen „standardized mean difference“ [SMD] 0,10 und 0,16) sowie von Stresserleben (SMD 0,24, 95 %-Konfidenzintervall[KI] 0,19–0,28), gleichzeitig aber auch eine geringe Abnahme der Lebensqualität und des mentalen Wohlbefindens (statistisch signifikante SMDs zwischen 0,08 und 0,10; [25]). Auch in dieser Population zeigte sich, dass eine weibliche Geschlechtszugehörigkeit mit einem größeren Risiko eines ungünstigen peripandemischen Verlaufs verbunden war [25].

Analog zu internationalen Daten [15, 29] zeigte sich generell in Deutschland eine deutlich stärkere peripandemische Beeinträchtigung der psychischen Gesundheit bei *Frauen* [11]. Die Lebenszufriedenheit der Frauen variierte dabei in Abhängigkeit der (Nicht‑)Verfügbarkeit von Kinderbetreuungsangeboten bzw. Schulöffnungen, während dies auf die Lebenszufriedenheit der Männer keinen nachweisbaren Einfluss hatte. Dies deutet auf eine Mehrbelastung der Frauen durch familiäre Care-Arbeit hin [11].

Auch bei *Gesundheitspersonal* mit und ohne PatientInnenkontakt, d. h. ärztlichem, Pflege- und medizinisch-technischem Personal, findet sich in der VOICE-Studie während der COVID-19-Pandemie eine größere psychische Belastung durch Angstsymptome und Depressivität im Vergleich zum präpandemischen Zeitraum, wobei hier jeweils ca. 20 % der Befragten auffällige Screeningwerte aufwiesen (GAD-2 bzw. PHQ-2), gleichzeitig jedoch in der Frühphase der Pandemie eine geringere Ausprägung im Vergleich zur Allgemeinbevölkerung [20]. Insbesondere bei Pflegepersonal wurde im Verlauf der Pandemie eine erhöhte Belastung durch „moral distress“ beobachtet, der durch Nichtbefolgen(können) eigener moralischer Standards entsteht und vor der Pandemie hauptsächlich aus intensiv- und notfallmedizinischen Settings berichtet wurde. Als protektive Faktoren wurden in dieser Personengruppe ein höheres Kohärenzgefühl, Optimismus sowie soziale Unterstützung insbesondere innerhalb des Arbeitsteams identifiziert [8].

Für *vorbestehende psychische Erkrankungen* wurde in der German National Cohort (NAKO) keine eindeutig erhöhte Vulnerabilität beobachtet [27]. Insbesondere in der Frühphase der Pandemie sind die Befunde innerhalb wie über unterschiedliche Diagnosegruppen hinweg heterogen und deuten teilweise auf eine geringere Zunahme von Angstsymptomatik und Depressivität bei Menschen mit vorbestehenden psychischen Erkrankungen hin als bei psychisch Gesunden [14, 15, 27]. Daten des Sozio-oekonomischen Panels (SOEP) zeigen jedoch, dass Personen mit zu Beginn der Pandemie bestehender Depression oder Depression in der Vorgeschichte im weiteren Pandemieverlauf mehr Depressivität, Angstsymptomatik und Einsamkeit erlebten als Nichtbetroffene [3].

## Individuelle und gesamtgesellschaftliche Resilienzfaktoren

Obwohl also im Durchschnitt für die COVID-19-Pandemie ein relevanter Einfluss auf die psychische Gesundheit der Allgemeinbevölkerung zu beobachten war und einige Personengruppen besonders stark betroffen waren, zeigt sich bei genauerer Betrachtung der *Verläufe*, dass diese sowohl quantitativ wie auch qualitativ durchaus unterschiedlich ausgeprägt sind.

Auch ist die Datenlage *nicht eindeutig, was die negative Beeinträchtigung* der psychischen Gesundheit anbelangt. Bspw. wurde in der LORA(Longitudinal Resilience Assessment)-Längsschnittstudie [1] bei psychisch gesunden jungen Erwachsenen zu Beginn der Pandemie im Schnitt eine Besserung des psychischen Befindens beobachtet bei gleichzeitiger Abnahme von Alltagsstressoren („daily hassles“), wobei jeweils 8 % der Teilnehmenden entweder eine stetige Verschlechterung des psychischen Wohlbefindens oder eine Rückkehr zum Ausgangsniveau nach anfänglicher Beeinträchtigung zeigten. Etwa 84 % zeigten hingegen keine Verschlechterung oder sogar eine Verbesserung [1].

Metaanalysen internationaler peripandemischer Beobachtungsstudien [24] konnten zeigen, dass *resiliente Verläufe* (d. h. ohne schwerwiegende Beeinträchtigung des psychischen Wohlbefindens oder mit rascher Rückkehr zum Ausgangsniveau nach anfänglicher Beeinträchtigung) der Regelfall waren: Analog zu präpandemischen Daten beträgt in bis Juli 2022 identifizierten Beobachtungsstudien die Prävalenz resilient-stabiler Verläufe auch in Zeiten der Pandemie ca. zwei Drittel (66 %), diejenige moderat-stabiler Verläufe ca. ein Viertel (27 %), während „recovery“ (d. h. eine rückläufige Belastung nach initialer Belastungszunahme) im Mittel zu 13 % berichtet wurde. Protrahiert-ungünstige („delayed“) und gleichbleibend-chronische Verläufe wurden dagegen am seltensten, d. h. zu jeweils ca. 11 % identifiziert [24]. Da die Mehrzahl der eingeschlossenen Studien keine präpandemischen Baseline-Daten berichten, kann hier eine Unterschätzung der Prävalenz von Recovery-Verläufen nicht ausgeschlossen werden.

Als *hilfreiche psychosoziale Faktoren für einen resilienten Verlauf* insbesondere in der Frühphase der Pandemie wurden u. a. in der längsschnittlichen DynaCORE-L-Studie ein positiver COVID-19-bezogener Bewertungsstil („positive appraisal style“), das wahrgenommene Vorhandensein sozialer Unterstützung sowie die Fähigkeit zu einer adaptiven kognitiven Emotionsregulation identifiziert [1, 15, 28]. Weitere wichtige psychosoziale Resilienzfaktoren umfassen neben sozialer Unterstützung und flexiblen kognitiv-behavioralen Strategien der Emotionsregulation und Problembewältigung hilfreiche Kontrollüberzeugungen wie bspw. eine hohe Selbstwirksamkeit, eine hohe selbstberichtete Resilienz, dispositioneller Optimismus sowie Sinnhaftigkeit im Leben, im Sinne eines ausgeprägten Kohärenzgefühls oder Spiritualität [24].

Insgesamt stellt eine *hinreichende regulatorische Flexibilität*, d. h. der situativ angepasste Einsatz kognitiv-emotionaler Bewältigungstechniken oder -mechanismen, übergreifend ein wesentliches Konstrukt dar, welches verschiedene Resilienzfaktoren und -mechanismen integriert und die eigentliche Resilienz, d. h. die Aufrechterhaltung oder rasche Widerherstellung der psychischen Gesundheit während oder nach stressvollen Lebensumständen [13], erst ermöglicht [9, 28]. Demzufolge sind Problemlösetechniken, Regulations- oder Copingmechanismen nicht per se hilfreich oder nicht, vielmehr ist deren Passung mit dem jeweiligen Stressor entscheidend [5]. Eine resiliente Antwort erfordert insoweit eine hinreichend zutreffende Bewertung der Situation sowie gleichzeitig die Verfügbarkeit eines Repertoires geeigneter Bewältigungsstrategien [5].

Auch auf* gesamtgesellschaftlicher Ebene* zeigt die COVID-19-Forschung einige für das mentale Wohlbefinden relevante Resilienzfaktoren, u. a. die wahrgenommene kollektive Selbstwirksamkeit („perceived collective efficacy“; [26]) oder der gesellschaftliche Zusammenhalt („social cohesiveness“), soziale Verbundenheit und soziales Engagement [2]. Konsistente Befunde einer höheren Beeinträchtigung des Wohlbefindens bei niedrigerem sozioökonomischem Status, mit institutionellem Vertrauen als signifikanter Moderator (u. a. [16, 22]), zeigen den Stellenwert sozialer Sicherheit und der Förderung der Teilhabe benachteiligter Gruppen zur Minimierung von sozialer und gesundheitlicher Ungleichheit („health inequity“; [4]).

## Schlussfolgerung

Im Zuge einer gesamtgesellschaftlichen Krisenlage wie der COVID-19-Pandemie sind Konsequenzen für das psychische Wohlergehen erwartbar, was sich kurzfristig in erhöhter psychischer Belastung während der ersten COVID-19-Welle bzw. des ersten Lockdowns, aber auch in einer schnellen Erholung nach Abklingen der ersten Pandemiewelle im Sommer 2020 zeigte, gefolgt von einem erneuten stetigen Anstieg im Zuge des zweiten Lockdowns („acute stressor“, „recovery“ und „pandemic fatigue effects“ [10]). Die Daten zu klinisch relevanten Symptomausprägungen beruhen dabei i. d. R. auf hoch sensitiven Screeningfragebögen ohne weitere Diagnosesicherung. *Weite Teile der Allgemeinbevölkerung* zeigten *resiliente Verläufe* [10, 24]. Gleichzeitig waren einzelne Gruppen wie Kinder und Jugendliche, Frauen und sozioökonomisch Benachteiligte eindeutig stärker betroffen.

Die Datenlage zum Verlauf der psychischen Gesundheit während der COVID-19-Pandemie in Deutschland reicht derzeit bis einschließlich zur ersten Jahreshälfte 2022. Der weitere Verlauf kann nicht losgelöst von weiteren Krisen, wie dem Angriffskrieg Russlands auf die Ukraine seit Anfang 2022, anderen geopolitischen Krisen und der Klimakrise gesehen werden.

Neben der Förderung der individuellen Resilienz sind auch soziale und gesellschaftliche Resilienzfaktoren relevante Ansatzpunkte der „pandemic preparedness“ [26]: Eine geringere Einsamkeit, eine höhere kollektive Wirksamkeit („collective efficacy“) und allgemein eine bessere soziale Kohäsion über gesellschaftliche Gruppen hinweg sind nachweisbar förderlich für die Aufrechterhaltung der mentalen Gesundheit in und nach gesamtgesellschaftlichen Krisen [12]. Programme zur Förderung der sozialen Kohäsion, die also die soziale Verbundenheit und Solidarität zwischen unterschiedlichen gesellschaftlichen Gruppen, Individuen und Institutionen adressieren, Integration und Teilhabe auch marginalisierter Gruppen unterstützen, institutionelles Vertrauen stärken, sozioökonomische Härten abfedern und Chancengleichheit anstreben sowie für gesunde Lebensumwelten auf lokaler Ebene sorgen, sind geeignet, mittels gesellschaftliche Resilienz („community resilience“) mittelbar auch die individuelle psychische Resilienz zu fördern [12].

## Fazit für die Praxis

- Die COVID-19-Pandemie war für weite Teile der Bevölkerung mit zumindest vorübergehenden Beeinträchtigungen des psychischen Wohlbefindens im Sinne einer erhöhten Angstsymptomatik und Depressivität sowie einer verminderten Lebenszufriedenheit verbunden. Allerdings waren diese Beeinträchtigungen in verschiedenen Gruppen unterschiedlich stark ausgeprägt. Gesicherte Vulnerabilitätsfaktoren sind: weibliche Geschlechtszugehörigkeit, jüngeres Alter sowie finanzielle Sorgen.
- In der Mehrzahl der Fälle waren resiliente Verläufe beobachtbar. Wesentliche individuelle Resilienzfaktoren sind ein positiver Bewertungsstil, soziale Unterstützung und Selbstwirksamkeitserleben. Gesamtgesellschaftliche Faktoren betreffen den sozialen Zusammenhalt und das Vertrauen in Institutionen.
- Diese Erkenntnisse liefern konkrete Ansatzpunkte zur Vorbereitung auf künftige Pandemien („pandemic preparedness“).

## References

[1] Ahrens KF, Neumann RJ, Kollmann B et al (2021) Impact of COVID-19 lockdown on mental health in germany: longitudinal observation of different mental health trajectories and protective factors. Transl Psychiatry 11:1–10. 10.1038/s41398-021-01508-2 34282129 10.1038/s41398-021-01508-2 PMC8287278

[2] Banks J, Fancourt D, Xu X (2021) Mental health and the COVID-19 pandemic. In: Helliwell JF, Layard R, Sachs J, De Neve J‑E (Hrsg) World Happiness Report 2021. Sustainable Development Solutions Network, powered by the Gallup World Poll data, New York, S 109–130

[3] Benke C, Asselmann E, Entringer TM, Pané-Farré CA (2022) The role of pre-pandemic depression for changes in depression, anxiety, and loneliness during the COVID-19 pandemic: Results from a longitudinal probability sample of adults from Germany. Eur Psychiatr 65:e76. 10.1192/j.eurpsy.2022.2339 10.1192/j.eurpsy.2022.2339 PMC9706309 36325825

[4] Blume M, Bartig S, Wollgast L et al (2024) Determinants of Mental Health Inequalities Among People With Selected Citizenships in Germany. Int J Public Health 69:1607267. 10.3389/ijph.2024.1607267 39258269 10.3389/ijph.2024.1607267 PMC11383781

[5] Bonanno GA, Burton CL (2013) Regulatory Flexibility: An Individual Differences Perspective on Coping and Emotion Regulation. Perspect Psychol Sci 8:591–612. 10.1177/1745691613504116 26173226 10.1177/1745691613504116

[6] Easterlin RA, O’Connor KJ (2023) Three years of COVID-19 and life satisfaction in Europe: A macro view. Proc Natl Acad Sci U S A 120:e2300717120. 10.1073/pnas.2300717120 37126673 10.1073/pnas.2300717120 PMC10175845

[7] Entringer T, Kröger H, Schupp J et al (2020) Psychische Krise durch Covid-19? Sorgen sinken, Einsamkeit steigt, Lebenszufriedenheit bleibt stabil. SOEPpapers on Multidisciplinary Panel Data Research 1087, DIW, Berlin

[8] Erim Y, Geiser F, Beschoner P et al (2024) Arbeitsplatzbezogenes Belastungserleben und psychische Gesundheit der Beschäftigten im Gesundheitswesen während der COVID-19-Pandemie: Risiko- und Schutzfaktoren aus der VOICE-Studie. Bundesgesundheitsbl 67:1248–1255. 10.1007/s00103-024-03954-x 10.1007/s00103-024-03954-x PMC11549185 39331174

[9] Gilan D, Müssig M, Hahad O et al (2021in) Protective and Risk Factors for Mental Distress and Its Impact on Health-Protective Behaviors during the SARS-CoV‑2 Pandemic between March 2020 and March 2021 in Germany. Int J Environ Res Public Health 18:9167. 10.3390/ijerph18179167 34501756 10.3390/ijerph18179167 PMC8431087

[10] Godara M, Rademacher J, Hecht M et al (2023) Heterogeneous Mental Health Responses to the COVID-19 Pandemic in Germany: An Examination of Long-Term Trajectories, Risk Factors, and Vulnerable Groups. Healthcare 11:1305. 10.3390/healthcare11091305 37174848 10.3390/healthcare11091305 PMC10177770

[11] Huebener M, Waights S, Spiess CK (2024) Well-Being Throughout the COVID-19 Pandemic in Germany: Gendered Effects of Daycare and School Closures. SSRN Journal. 10.2139/ssrn.4788221

[12] Jewett RL, Mah SM, Howell N, Larsen MM (2021) Social Cohesion and Community Resilience During COVID-19 and Pandemics: A Rapid Scoping Review to Inform the United Nations Research Roadmap for COVID-19 Recovery. Int J Health Serv 51:325–336. 10.1177/0020731421997092 33827308 10.1177/0020731421997092 PMC8204038

[13] Kalisch R, Müller MB, Tüscher O (2015) A conceptual framework for the neurobiological study of resilience. Behav Brain Sci 38:e92. 10.1017/S0140525X1400082X 25158686 10.1017/S0140525X1400082X

[14] Kunzler AM, Lindner S, Röthke N et al (2023) Mental Health Impact of Early Stages of the COVID-19 Pandemic on Individuals with Pre-Existing Mental Disorders: A Systematic Review of Longitudinal Research. IJERPH 20:948. 10.3390/ijerph20020948 36673705 10.3390/ijerph20020948 PMC9858748

[15] Kunzler AM, Röthke N, Günthner L et al (2021) Mental burden and its risk and protective factors during the early phase of the SARS-CoV‑2 pandemic: systematic review and meta-analyses. Global Health 17:34. 10.1186/s12992-021-00670-y 33781283 10.1186/s12992-021-00670-y PMC8006628

[16] Lee S (2022) Subjective Well-being and Mental Health During the Pandemic Outbreak: Exploring the Role of Institutional Trust. Res Aging 44:10–21. 10.1177/0164027520975145 33234059 10.1177/0164027520975145

[17] Mata J, Wenz A, Rettig T et al (2021) Health behaviors and mental health during the COVID-19 pandemic: A longitudinal population-based survey in Germany. Soc Sci Med 287:114333. 10.1016/j.socscimed.2021.114333 34455337 10.1016/j.socscimed.2021.114333 PMC8479385

[18] Mauz E, Eicher S, Peitz D et al (2022) Mental health of the adult population in Germany during the COVID-19 pandemic. Rapid Rev. 10.25646/9537 10.25646/9537.2 PMC8832373 35585856

[19] Mauz E, Walther L, Junker S et al (2023) Time trends in mental health indicators in Germany’s adult population before and during the COVID-19 pandemic. Front Public Health. 10.3389/fpubh.2023.1065938 36908429 10.3389/fpubh.2023.1065938 PMC9995751

[20] Morawa E, Schug C, Geiser F et al (2021) Psychosocial burden and working conditions during the COVID-19 pandemic in Germany: The VOICE survey among 3678 health care workers in hospitals. J Psychosom Res 144:110415. 10.1016/j.jpsychores.2021.110415 33743398 10.1016/j.jpsychores.2021.110415 PMC7944879

[21] Ravens-Sieberer U, Devine J, Napp A‑K et al (2023) Three years into the pandemic: results of the longitudinal German COPSY study on youth mental health and health-related quality of life. Front Public Health. 10.3389/fpubh.2023.1129073 37397777 10.3389/fpubh.2023.1129073 PMC10307958

[22] Reed H, Thapar A, Riglin L et al (2024) The unequal impacts of the COVID-19 pandemic on young adults’ mental health. Predictors of vulnerability and resilience using longitudinal birth cohort data in the UK. J Adolesc. 10.1002/jad.12400 39205604 10.1002/jad.12400 PMC11701400

[23] Robinson E, Sutin AR, Daly M, Jones A (2022) A systematic review and meta-analysis of longitudinal cohort studies comparing mental health before versus during the COVID-19 pandemic in 2020. J Affective Disord 296:567–576. 10.1016/j.jad.2021.09.098 10.1016/j.jad.2021.09.098 PMC8578001 34600966

[24] Schäfer SK, Kunzler AM, Kalisch R et al (2022) Trajectories of resilience and mental distress to global major disruptions. Trends Cogn Sci 26:1171–1189. 10.1016/j.tics.2022.09.017 36302711 10.1016/j.tics.2022.09.017 PMC9595401

[25] Schäfer SK, Lindner S, Kunzler AM et al (2023) The mental health impact of the COVID-19 pandemic on older adults: a systematic review and meta-analysis. Age Ageing 52:afad170. 10.1093/ageing/afad170 37725975 10.1093/ageing/afad170

[26] Schäfer SK, Supke M, Kausmann C et al (2024) A systematic review of individual, social, and societal resilience factors in response to societal challenges and crises. Commun Psychol 2:1–22. 10.1038/s44271-024-00138-w 39369098 10.1038/s44271-024-00138-w PMC11455977

[27] Stein J, Pabst A, Berger K et al (2024) Mental health of individuals with pre-existing mental illnesses at the beginning of the COVID-19 pandemic: results of the German National Cohort (NAKO). Front Public Health. 10.3389/fpubh.2024.1451631 39377001 10.3389/fpubh.2024.1451631 PMC11456423

[28] Veer IM, Riepenhausen A, Zerban M et al (2021) Psycho-social factors associated with mental resilience in the Corona lockdown. Transl Psychiatry 11:1–11. 10.1038/s41398-020-01150-4 33479211 10.1038/s41398-020-01150-4 PMC7817958

[29] Witteveen AB, Young SY, Cuijpers P et al (2023) COVID-19 and common mental health symptoms in the early phase of the pandemic: An umbrella review of the evidence. PLoS Med 20:e1004206. 10.1371/journal.pmed.1004206 37098048 10.1371/journal.pmed.1004206 PMC10129001

[30] Wolf K, Schmitz J (2024) Scoping review: longitudinal effects of the COVID-19 pandemic on child and adolescent mental health. Eur Child Adolesc Psychiatry 33:1257–1312. 10.1007/s00787-023-02206-8 37081139 10.1007/s00787-023-02206-8 PMC10119016

[31] Zoellner F, Erhart M, Napp A‑K et al (2024) Risk and protective factors for mental health problems in children and adolescents during the COVID-19 pandemic: results of the longitudinal COPSY study. Eur Child Adolesc Psychiatry. 10.1007/s00787-024-02604-6 39470791 10.1007/s00787-024-02604-6 PMC12198059

